# Retinal Infarction: A Pilot Study on the Efficacy and Safety of Intravenous Thrombolysis and Underlying Aetiologies

**DOI:** 10.3390/life12081279

**Published:** 2022-08-22

**Authors:** Sonja Schönecker, Johannes Wischmann, Dennis C. Thunstedt, Katharina Feil, Marc J. Mackert, Siegfried Priglinger, Lars Kellert

**Affiliations:** 1 Department of Neurology, Ludwig-Maximilians-Universität München, LMU, 81377 Munich, Germany; 2 Department of Neurology & Stroke, Eberhard-Karls University, 72074 Tübingen, Germany; 3 Department of Ophthalmology, Ludwig-Maximilians-Universität München, LMU, 81377 Munich, Germany

**Keywords:** non-arteritic central retinal artery occlusion, IV thrombolysis, microembolism, endarterectomy

## Abstract

**Background:** Treatment of non-arteritic central retinal artery occlusion is still inconsistent. Therefore, the current study aimed to evaluate the efficacy of intravenous thrombolysis (IVT) and describe the prevalence of co-occurring ischemic brain lesions in patients with acute visual loss due to ischemia. **Methods:** We analysed 38 consecutive patients with acute visual loss between January 2015 and June 2020. Patients presenting within 4.5 h of symptom onset without any contraindication were treated with IVT. Patients underwent neurologic and ophthalmologic examination and diagnostic workup for the underlying aetiology. Follow-up was performed after 3 and 12 months. **Results:** Patients treated with IVT had a significantly better functional outcome at discharge compared to patients treated conservatively. No additional ischemic brain lesions were detected (0 of 38). Three patients had extracranial carotid artery stenosis ≥50%. Atrial fibrillation was present in four patients, three of whom already received oral anticoagulation. In the remaining 31 patients no embolic source was detected. However, the number of plaques were rated mild to moderate. Within three months, one patient developed transient visual loss while another suffered a contralateral transient ischemic attack. **Conclusions:** IVT may represent a safe and effective treatment option in patients with isolated visual loss due to ischemia. The aetiology was atherosclerotic burden rather than embolism caused by carotid stenosis or atrial fibrillation, bringing the current diagnostic procedure and therapy into question. Randomized trials are necessary to evaluate the efficacy and safety of IV thrombolysis and clarify the aetiology of isolated visual loss due to ischemia.

## 1. Introduction

Central retinal artery occlusion (CRAO) without underlying giant cell arteritis, i.e., non-arteritic central retinal artery occlusion (NA-CRAO) is defined by sudden profound visual loss due to ischemia to the inner retinal layer and the surface of the optic nerve [1,2]. The incidence is estimated to range between 1 and 2 in 100,000 persons [3] and it mostly affects patients over the age of 60 [4]. Men are slightly more frequently affected than women [5]. It is considered a form of acute stroke that is most commonly caused by embolism to the central retinal artery or branch retinal arteries from plaques in the ipsilateral internal carotid artery, the aortic arch or the heart [6]. Cardiovascular risk factors such as obesity, hypertension, smoking, hypercholesterolemia, and diabetes are highly prevalent [7,8] and are associated with an increased risk of future cardiovascular and cerebrovascular events [9,10]. Furthermore, NA-CRAO patients are more likely to have cardiac arrhythmias, atrial fibrillation, cardiac valvular disease, or heart failure [11]. In addition to the exclusion of giant cell arteritis by means of blood sedimentation, C-reactive protein and ultrasound of the vessels supplying the brain, an ophthalmological evaluation including a dilated funduscopic examination is performed diagnostically [12]. To date, there are no evidence-based treatments for NA-CRAO. However, given the poor prognosis after NA-CRAO with 80% of patients showing a visual acuity of “count fingers” or worse at follow-up [13] an attempt at treatment is usually made. However, clinical management has been heterogeneous in recent decades. Conservative approaches that have been used in an effort to restore vision include ocular massage, anterior chamber paracentesis, nootropics, oxygen and acetazolamide. However, recent studies were not able to detect a beneficial effect of these treatments compared to placebo and even suggested a reduced visual recovery rate compared to the natural course of the disease [14,15]. Furthermore, the effect of intra-arterial thrombolysis has been evaluated in NA-CRAO. However, the published results have been inconsistent [16]. While some case series suggest that intra-arterial thrombolysis may improve visual outcomes [17], the only randomized controlled study (EAGLE) was stopped prematurely as no superiority of intra-arterial thrombolysis compared to standard of care treatment was detectable [7]. However, as no patient in the EAGLE study received therapy earlier than 4 h after symptom onset the results are not generalizable to earlier time points and the time frame of 4.5 h typically used in stroke research remains untested. In recent years, intravenous thrombolysis (IVT) has gradually come to the fore. For years IVT has been used empirically to treat NA-CRAO. A patient-level meta-analysis of observational studies showed a beneficial effect of IVT at 4.5 h or earlier after symptom onset compared to the natural course of the disease or conservative treatment [14] and these results could be replicated in a recent updated meta-analysis including four modern cohorts of acute CRAO [18]. However, fully powered efficacy trials evaluating the utility of IVT are still missing. The current study aimed to evaluate the efficacy of IVT and describe the prevalence of cooccurring and subsequent ischemic stroke and the underlying aetiologies in patients with acute visual loss due to ischemia and thereby provide clarity regarding the practical therapeutic approach.

## 2. Methods

### 2.1. Ethics Statement

The study was performed according to the declaration of Helsinki (1991). Retrospective analysis of data has been approved by the local ethics committee (17-074, 23 February 2017; Medical Faculty, Ludwig-Maximilians-Universität München, Munich, Germany). Due to the retrospective nature of the study and anonymised data analysis, no consent to participate was required. 

#### Study Population

We analysed a consecutive set of 38 patients presenting at our emergency department with the main complaint of acute visual loss between January 2015 and June 2020. Patients who presented to the eye clinic were later transferred to the neurology department. The diagnosis of retinal artery occlusion based on an ophthalmological examination including best corrected visual acuity (visual acuity), a swinging flashlight test, a complete ophthalmological examination of the anterior segments of the eye and a binocular fundus examination in miosis, as well as an applanatory intraocular pressure measurement according to Goldmann. All patients underwent neurologic evaluation including modified Rankin scale (mRS, Table 1) [19] at admission and emergency computed tomography (CT) and CT angiography (CTA). In analogy to acute cerebral ischemic stroke, patients presenting within 4.5 h of symptom onset without any contraindication to IVT (e.g., detection of haemorrhage on cerebral CT scan, use of anticoagulation, etc.) received recombinant tissue Plasminogen Activator (rtPA, Actilyse^®^, Boehringer Ingelheim, 55218 Ingelheim am Rhein, Germany) at a dose of 0.9 mg/kg (10% administered as bolus and the remainder infused over 1 h). All patients were admitted to the Stroke Unit of the Department of Neurology, LMU Munich for intensive neuromonitoring and diagnostic workup including echocardiogram, cardiac monitoring, transthoracic and transoesophageal echocardiography respectively, ultrasonography of intra- and extra-cranial vessels and basic blood tests (including erythrocyte sedimentation rate, serum chemistry, complete blood count, activated thromboplastin time and prothrombin time). The extent of plaques in extracranial vessels at ultrasonography was rated visually and semi-quantitatively on a four-item scale (0 = no plaques, 1 = mild, 2 = moderate, 3 = severe). All but two patients with transient visual loss (TVL) underwent a detailed ophthalmologic examination including assessment of visual acuity, pupils, anterior segment, ocular motility and fundoscopic examination. Additionally, magnetic resonance imaging (MRI) of the brain was performed in 18 patients (11 with NA-CRAO, two with branch retinal artery occlusion (BRAO) and five with TVL) during the inpatient stay. Moreover, CSF analysis, vasculitis markers, thrombophilia testing and cancer screening was performed where deemed necessary. In all patients having received IVT, follow-up cerebral imaging by either CT or MRI was performed after 24 h. Medical history, any diagnosis, acute procedure or change in medication during the inpatient stay as well as mRS at discharge was recorded. An insertable cardiac monitor was implanted in 18 patients during the inpatient stay. 

Follow-up at 90 days (all patients) and 12 months (23 patients) was either performed in the outpatient department for vascular neurology or by a stringently performed standardized telephone interview. Medical history, adverse events and mRS were recorded. When evaluating stroke risk clinically symptomatic strokes or transient ischemic attacks were counted. The mRS is the most widely used scale to express the functional independence of stroke patients in everyday life. In most clinical stroke studies, mRS is the primary endpoint. Therefore, we also determined it in our patients on admission, after 24 h and in the 3-month follow-up. An mRS of 0–2 is considered a good outcome after a stroke.

### 2.2. Statistical Analysis

Data were analysed using IBM SPSS Statistics for Windows (Version 26.0, IBM Armonk, NY, USA). Non-dichotomized mean scores of demographic and clinical data were compared across the three groups (TVL, NA-CRAO/BRAO with standard of care treatment, NA-CRAO treated with IV thrombolysis) via Kruskal–Wallis test and post hoc Bonferroni corrected Mann–Whitney tests (number of comparisons = 3, adjusted *p*-value < 0.017). Chi-square analysis was used to check for differences in dichotomized variables across all groups. 

## 3. Results

A total of 38 patients presented between January 2015 and June 2020 with acute visual loss at our emergency department. Thereof, 19 patients were diagnosed with non-arteritic central retinal occlusion (NA-CRAO), 6 with branch retinal artery occlusion (BRAO) and 13 with transient visual loss (TVL). Nine NA-CRAO patients were treated with IVT, one of whom developed an asymptomatic intraparenchymal haemorrhage. In terms of vascular risk factors, 68.4% had hypertension (permanent increase of systolic blood pressure over 140 mmHg, and a diastolic increase to more than 90 mmHg, independent of the situation), 13.2% diabetes (chronic, metabolic disease characterized by elevated levels of blood glucose), 10.5% a positive family history for cardiovascular events (cardiovascular events in a first degree relative below the age of 65), 28.9% hypercholesterolemia (raised cholesterol levels) and 21.1% of patients were active smokers. The mean body mass index was 26.8. Demographics and clinical scores of the study sample are provided in Table 2.

Patient groups did not differ in terms of age, gender, side of symptoms and aetiology. Significant group differences regarding the occurrence of a positive family history were detected, however, post hoc Bonferroni corrected tests did not show significant pairwise differences. The remaining cardiovascular risk factors did not differ between groups. 

### 3.1. Underlying Aetiology

Atrial fibrillation was present in four patients (10.5%), two of whom had sufficient oral anticoagulation while one patient’s international normalized ratio ranged at a borderline value of 1.9. Large artery sclerosis defined by ipsilateral carotid stenosis ≥50% was present in three patients (7.9%). The aetiology of the remainder of patients was classified as embolic stroke of undetermined source (ESUS). A cooccurring cerebral infarction could not be detected in any patient. One patient with TVL (2.6%) classified as ESUS already had a history of NA-CRAO while two patients with TVL, one of whom was classified as ESUS while the other had a stenosis ≥50% showed older silent ischemia on MRI (5.3%). 

### 3.2. Clinical Outcome

As expected, visual acuity at admission was best in the group of TVL patients and was 0.65 ± 0.37 on average. In contrast, the majority of NA-CRAO and BRAO patients had a visual acuity of “counting fingers” or worse. Compared to patients with TVL and NA-CRAO and BRAO treated conservatively respectively, patients treated with IVT had a significantly larger difference between mRS at admission and discharge (*p* = 0.006) (Table 3).

All patients were followed for three months. In 24 patients, additional follow-up data at 12 months were available. One patient with TVL and known atrial fibrillation experienced another TVL within three months under ongoing oral anticoagulation, while another whose aetiology was classified as ESUS had a transient ischemic attack, contralateral to the TVL. In one patient a myocardial infarction occurred two months after NA-CRAO and in another patient with NA-CRAO non-vascular death occurred. No patient followed for 12 months experienced a cardiovascular event between the third and twelfth month. Interestingly none of the three patients with large artery sclerosis, two of which were followed for 12 months, experienced another cardiovascular event. 

In three out of 18 patients having received an insertable cardiac monitor, atrial fibrillation could be detected within three months. In the time interval between the third and twelfth month no further case of atrial fibrillation could be detected. 

## 4. Discussion

To date management of NA-CRAO still remains inconsistent. However, recent studies have provided evidence for the superiority of IVT compared to standard of care treatment [18,20,21]. The current study adds to these data and suggests IVT to be feasible, safe and lead to a better clinical outcome. However, due to the retrospective uncontrolled design of our study the results must be interpreted with caution. The difference between mRS at admission and discharge of NA-CRAO patients treated with IVT was not only higher compared to TVL patients but also to NA-CRAO/BRAO patients treated conservatively. The effectiveness of IVT is further underlined by the fact that the group of patients treated conservatively also included BRAO patients, who tend to have a better prognosis [6]. To date, there have been no adequate randomized placebo-controlled interventional studies of IVT in NA-CRAO/BRAO. However, three randomized trials evaluating the efficacy and safety of IV thrombolysis compared to placebo in patients with acute visual loss due to ischemia are currently underway: THEIA (A Phase III Randomized, Blind, Double Dummy, Multicentre Study Assessing the Efficacy and Safety of IV Thrombolysis [Alteplase] in Patients with Acute Central Retinal Artery Occlusion), REVISION (Early Reperfusion Therapy with Intravenous Alteplase for Recovery of Vision in Acute Central Retinal Artery Occlusion) and Ten-CRAOS (Tenecteplase in Central Retinal Artery Occlusion Study). 

The foremost argument that has been raised in the past against the use of IVT is the risk of haemorrhagic complications, especially intracerebral haemorrhage. However, in our study only one patient developed an asymptomatic intraparenchymal haemorrhage. This is in line with previous studies showing a low risk of adverse events after IVT in NA-CRAO patients. To date there are no recorded cases of symptomatic intracranial haemorrhages in patients treated within 4.5 h after symptom onset and without concomitant anticoagulation [18,22]. Overall, in the absence of overt neurological manifestations the rate of IVT related adverts events in NA-CRAO patients can be expected to equal those in minor stroke, a condition in which adverse events related to IVT are exceedingly low (2% to 2.4%) [23,24,25]. 

Interestingly, in none of our patients could a cooccurring cerebral infarction be detected. This is in contrast to previous research that showed a cooccurrence rate of 24%, with only about a half of patients exhibiting cerebral neurological signs or symptoms [26,27]. This discrepancy is probably due to the fact that cerebral MRI was performed in only 18 of our 38 patients. However, none showed clinical signs of cerebral infarction and CT did not detect ischemic brain lesions either. The absence of cooccurring symptomatic cerebral infarction can be explained by the fact that arterial occlusions in the eye are most commonly due to microembolism [28]. Particles in the bloodstream are distributed along a radial gradient with large particles being more concentrated in the axial stream, while small particles are more concentrated in the peripheral stream (Fahraeus–Lindqvist effect) and are therefore preferentially distributed to the ophthalmic artery but seldom into the hemispheric circulation [27]. The major source of microembolism is plaque but not necessarily stenosis. This is in line with our data, as significant stenosis (≥50%) was present in only three patients while the amount of plaque was rated mild to moderate on average. This, however, leads to the question whether stenosis ≥50% in cases of isolated NA-CRAO should be rated as symptomatic and whether endarterectomy or carotid artery stenting which may represent a source of microembolization [29,30] are appropriate in these patients or whether plaque stabilizing therapies may be a more suitable treatment option. To date data supporting endarterectomy or carotid artery stenting in NA-CRAO are tenuous [31]. The fact that three out of four patients with atrial fibrillation developed acute visual loss due to ischemia under ongoing oral anticoagulation and one of these patients experienced a further TVL within the first three months after the primary event, leads to the question whether atrial fibrillation was indeed the aetiology in these cases. Thus, our study raises doubts about the embolic cause of retinal infarctions—following the usual definition of embolic sources such as carotid stenosis or atrial fibrillation. Although our results are based on single centre data with a small number of patients and a non-controlled observational design, putative implications could be far-reaching. If the aetiology of retinal infarctions would be more likely due to microembolism based on atherosclerotic burden than on embolic sources such as atrial fibrillation or higher-grade stenosis, this would emphasize the need for a change in diagnostic workup and secondary prevention. 

## 5. Conclusions

In our small study, IVT was a safe and effective treatment option in patients with isolated visual loss due to ischemia. The underlying aetiology was rather atherosclerotic burden than embolism caused by higher-grade carotid stenosis or atrial fibrillation, which questions the current diagnostic procedure and treatment and has implications for diagnostics, therapy and secondary prevention. Randomized trials will be needed to evaluate the efficacy and safety of IV thrombolysis and clarify the aetiology of isolated visual loss due to ischemia. 

## Figures and Tables

**Table 1:** Modified Rankin Scale.

Grade	Description
0	No symptoms at all
1	No significant disability despite symptoms: able to carry out all usual duties and activities
2	Slight disability: unable to carry out all previous activities but able to look after own affairs without assistance
3	Moderate disability: requiring some help, but able to walk without assistance
4	Moderately severe disability: unable to walk without assistance, and unable to attend to own bodily needs without assistance
5	Severe disability: bedridden, incontinent, and requiring constant nursing care and attention
6	Death *

* Commonly used in clinical trials.

**Table 2:** Demographic and clinical data.

	Total Population	TVL	NA-CRAO/BRAO	NA-CRAO	
		Standard of Care	Standard of Care	IV Thrombolysis	*p*-Value
	*n* = 38	*n* = 13	*n* = 16	*n* = 9	
Age	69.4 ± 11.5	65.2 ± 11.3	72.4 ± 13.2	70.2 ± 6.9	0.084
Female	31.6%	30.8%	43.8%	11.1%	0.241
right sided	63.2%	61.5%	75.0%	55.6%	0.684
ESUS	81.6%	69.2%	81.3%	100.0%	0.187
Cardioembolic	10.5%	15.4%	12.5%	0.0%	0.484
Macroangiopathic	7.9%	15.4%	6.3%	0.0%	0.400
Prior symptomatic stroke or TIA	2.6%	7.7%	0.0%	0.0%	0.372
Hypertension	68.4%	69.2%	62.5%	77.8%	0.730
Diabetes	13.2%	15.4%	18.8%	0.0%	0.395
Current smoking	21.1%	30.8%	12.5%	22.2%	0.461
Hypercholesterolemia	28.9%	30.8%	43.8%	0.0%	0.067
Positive family history	10.5%	30.8%	0.0%	0.0%	**0.014**
Body mass index	26.8 ± 4.2	27.1 ± 3.8	26.4 ± 4.5	27.0 ± 4.8	0.824
Plaque rating	1.6 ± 1.0	1.2 ± 1.0	1.8 ± 1.0	2.0 ± 0.9	0.157

Significantly different compared to TVL, NA-CRAO/BRAO—standard of care, NA-CRAO—IV thrombolysis. TVL transient visual loss; NA-CRAO non-arteritic central retinal artery occlusion; BRAO branch retinal artery occlusion; ESUS embolic stroke of undetermined source; mRS modified Rankin Scale.

**Table 3:** Clinical outcome parameters.

	Total Population	TVL	NA-CRAO/BRAO	NA-CRAO	
		Standard of Care	Standard of Care	IV Thrombolysis	*p*-Value
	*n* = 38	*n* =13	*n* = 16	*n* = 9	
mRS admission—discharge	0.3 ± 0.7	0.2 ± 0.6 ^c^	0.1 ± 0.6 ^c^	0.9 ± 0.9 ^a,b^	**0.006**
Cardiovascular events	7.9%	15.4%	6.3%	0.0%	0.400
Non-vascular death	2.6%	0.0%	6.3%	0.0%	0.494
detection of atrial fibrillation	7.9%	7.7%	6.3%	11.1%	0.914

Significantly different compared to ^a^ TVL, ^b^ NA-CRAO/BRAO—standard of care, ^c^ NA-CRAO—IV thrombolysis. TVL transient visual loss; NA-CRAO non-arteritic central retinal artery occlusion; BRAO branch retinal artery occlusion; ESUS embolic stroke of undetermined source; mRS modified Rankin Scale.

## Abbreviations

Non-standard Abbreviations and Acronyms: BRAO: branch retinal artery occlusion, ESUS: embolic stroke of undetermined source, NA-CRAO: non-arteritic central retinal artery occlusion, mRS: modified Rankin Scale, TVL: transient visual loss.
